# A case of fulminant myocarditis with full recovery after a 38‐h sustained asystole

**DOI:** 10.1002/ccr3.7722

**Published:** 2023-07-30

**Authors:** Tomohiro Akutsu, Akira Endo, Hiroyuki Sonobe, Keisuke Suzuki, Kiyoshi Murata, Yasuhiro Otomo

**Affiliations:** ^1^ Department of Acute Critical Care Medicine Tsuchiura Kyodo General Hospital Tsuchiura Japan; ^2^ Department of Acute Critical Care Medicine Matsudo General Hospital Matsudo Japan; ^3^ Department of Acute Critical Care and Disaster Medicine Tokyo Medical and Dental University Graduate School of Medicine Bunkyo‐ku Japan

**Keywords:** asystole, cardiac arrest, extracorporeal membrane oxygenation, fulminant myocarditis, intra‐aortic balloon pumping

## Abstract

**Key Clinical Message:**

Even if cardiac rhythm deteriorated to asystole in the clinical course of fulminant myocarditis, cardiac function may recover, and the patient may be discharged without brain damage, if circulation could be maintained by appropriate mechanical cardiac supports.

**Abstract:**

A 69‐year‐old man was diagnosed with fulminant myocarditis with circulatory collapse. His cardiac rhythm deteriorated to asystole on the second day; however, circulatory status was maintained through extracorporeal membrane oxygenation and intra‐aortic balloon pumping. After 38 h‐lasting asystole, his heart resumed beating. He was discharged without neurological deficits on Day 25.

## Graphical abstract

Vital and ECMO flow and time course of asystole from day 1 to day 4 of admission in the intensive care unit.

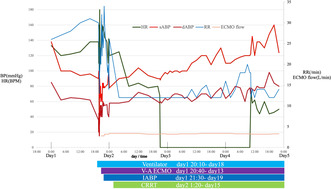

## 1 INTRODUCTION

Fulminant myocarditis is an uncommon, but severe cardiac inflammatory disease that can be fatal.^1^, ^2^ The disease is characterized by a severe and sudden onset marked by a rapid progressive deterioration that can occur within 2 or 3 days.^3^ The use of extracorporeal membrane oxygenation (ECMO) is common; therefore, its application in fulminant myocarditis has also increased.^4^ Several case reports indicated the use of mechanical circulation support (MCS) devices, including ECMO, which provides full circulatory support with time for the heart to recover.^5^, ^6^, ^7^ However, to our knowledge, there are no reports of a patient with fulminant myocarditis who subsequently recovered cardiac function after prolonged asystole.^5^, ^6^, ^7^, ^8^

## 2 CASE PRESENTATION

A 69‐year‐old man with no history of cardiac disease was transported to a previous hospital with a fever of 39°C and fainting. An electrocardiogram (ECG) showed ST‐segment elevation and elevated myocardial desensitization enzymes. Echocardiography revealed severe diffuse hypokinesis and pericardial effusion. Additionally, remarkable stenosis was not observed during coronary artery angiography. He was admitted to the previous hospital with suspected myocarditis. Three days after admission, his level of consciousness decreased, hepatobiliary enzymes increased, renal function worsened, and blood pressure decreased; thereafter, he was transferred to our hospital.

**FIGURE 1:**
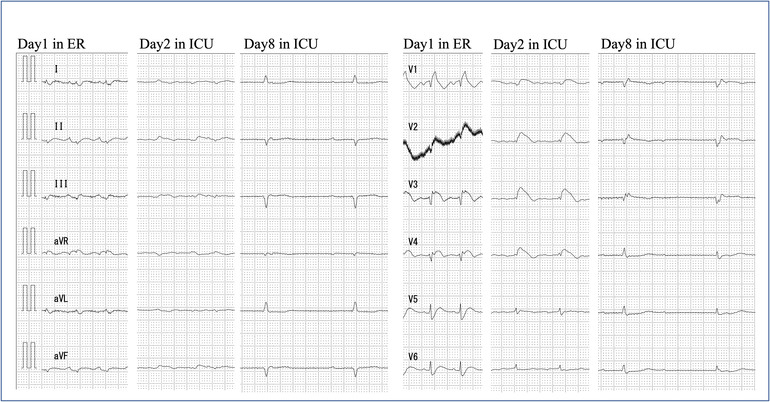
Twelve‐lead electrocardiogram (ECG) on Days 1,2, and 8. Day 1 in emergency room (ER): All inductions showed ST elevation. Day 2 in intensive care unit (ICU): ECG just before extracorporeal membrane oxygenation was introduced. Day 8 in intensive care unit (ICU): ECG showing recovery of cardiac function. ejection fraction at this time was about 15%.

**FIGURE 2:**
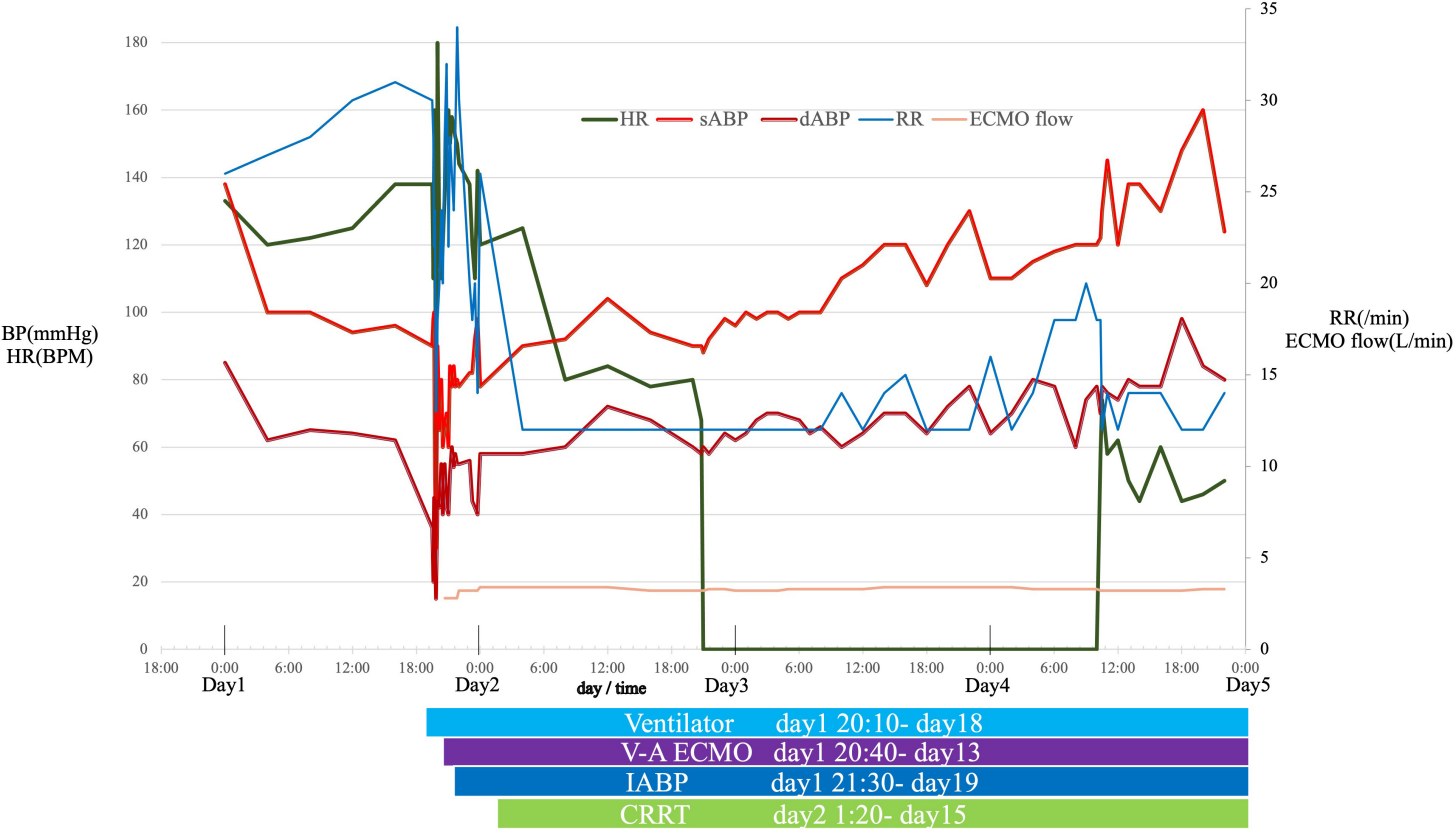
Vital sheet in the intensive care unit on Day 1‐Day 4. Heart rate (HR), systolic blood pressure (sBP), diastolic blood pressure (dBP), respiratory rare (RR), and extracorporeal membrane oxygenation (ECMO) flow in the intensive care unit on Day 1 to Day 4. Below, the horizontal axis are bars showing the start and end dates of ventilator, ECMO, intra‐aortic balloon pumping (IABP), and continuous renal replacement therapy (CRRT). The period when HR is 0 shows asystole, but blood pressure and ECMO flow are also maintained. Asystole lasted approximately 38 h.

On physical examination at our emergency department, his Glasgow Coma Scale score was 14 (eyes: 4, verbal: 4, motor: 6); blood pressure (BP): 112/90 mmHg; heart rate (HR): 132 beats per minute (bpm), regular with no catecholamine support; respirations: 27 breaths per minute, oxygen saturation: 96% with a 4 L oral mask, and temperature was 37.2°C. The laboratory data at the time of arrival are shown in Table 1. Overall, white blood cells, C‐reactive protein (CRP), and myocardial enzymes were prominently elevated. Troponin I levels were above 2000 ng/L. A 12‐lead ECG showed ST‐segment elevation in all guides (Figure 1). After admission to the intensive care unit (ICU), considering the possibility of a bacterial infection, appropriate treatment was initiated. On Day 2, his HR increased to 180 bpm and his systolic BP dropped to 60 mmHg. Echocardiography revealed a significant decrease in ejection fraction to approximately 10%. The patient then fell into pulseless electrical activity (PEA); cardiopulmonary resuscitation (CPR) occurred immediately, and the patient was resuscitated after administration of 1 mg of adrenaline. After 17 minutes of CPR, return of spontaneous circulation was achieved. Although circulation could be maintained with catecholamine support for several minutes, blood pressure gradually decreased. Approximately 60 min after the initial PEA, veno‐arterial ECMO (V‐A ECMO), from the right femoral vein to the left femoral artery was introduced. Intra‐aortic balloon pumping (IABP) was added. Intravenous high‐dose methylprednisolone therapy (1000 mg for 3 days) and immunoglobulin therapy (0.5 kg/kg for 2 days) were also initiated. On Day 4, ECG showed asystole; however, systolic BP was maintained at 80 mmHg under V‐A ECMO with a flow of approximately 3 L/min and IABP with an internal trigger mode. Circulatory dynamics were maintained; therefore, intensive care was continued. After 38 h of asystole, electrical activity was recorded on ECG with an HR of 50–60 bpm (Figure 2). He responded to a call, suggesting cerebral function was maintained to some level. Cardiac function gradually improved, and the patient was weaned off V‐A ECMO on Day 14. The cause of fulminant myocarditis in this case was unclear, as we did not perform magnetic resonance imaging or myocardial biopsy at our hospital. Laboratory data that screened for causative viruses also showed no significant findings. On Day 15, echocardiography showed an improved ejection fraction of approximately 60%. The patient was weaned from the IABP and extubated on Day 18. After extubation, consciousness continued without any obvious higher functional impairment. The patient's renal and hepatic functions completely recovered, and he was weaned from continuous renal replacement therapy. There were no central nervous system disorders, and his cerebral performance category was 1. There were no other complications, and the echocardiography just before discharge showed an ejection fraction of about 65%. On Day 25, the patient was transferred to another hospital. Blood samples were taken for IgG and IgM antibodies against enterovirus, adenovirus, parvovirus, influenza virus, herpes virus, cytomegalovirus, and EB virus; none showed high levels of the IgM antibody.

**TABLE 1:** Laboratory data from the emergency room.

Complete blood count date			Biochemistry date			Coagulation date			Arterial blood gas		
WBC	13,800	/μL	T‐Bil	0.74	mg/dL	PT (%)	83	%	pH	7.166	
Seg	87.2	%	AST	1315	IU/l	PT‐INR	1.12	mg/dL	pCO_2_	39.1	mmHg
Eo	1.2	%	ALT	484	IU/l	APTT	23.3	sec	pO_2_	417.1	mmHg
Baso	0.2	%	LDH	1993	IU/l	Fibrinogen	260	mg/dL	HCO_3_^−^	13.8	mmol/l
Mono	3.1	%	Γ‐GTP	101	IU/l	D‐dimer	2.6	μg/mL	B.E	−13.8	mmol/l
Lymph	8.3	%	CPK	8116	IU/l				Lactate	9.5	mmol/l
RBC	498	×10^4^/μL	CK‐MB	141	IU/l						
Hb	15.0	g/dL	Troponin T	>2000	ng/l						
Hct	42.9	%	BNP	562.2	Pg/mL						
PLT	6.1	×10^4^/μL	BUN	48.4	mg/dL						
			Cre	2.82	mg/dL						
			Na	128	mEq/l						
			Cl	97	mEq/l						
			K	4.1	mEq/l						
			Ca	7.6	U/l						
			Tp	5.2	g/dL						
			Alb	4.1	g/dL						
			CRP	0.18	mg/dL						

Abbreviations: Alb, Albumin; ALT, Alanine aminotransferase; APTT, Activated partial thromboplastin time; AST, Aspartate aminotransferase; B.E, Base excess; Baso, Basophil; BUN, Blood urea nitrogen; CPK, Creatine phosphokinase; Cre, Creatinine; CRP, C‐reactive protein; Direct‐ Bilirubin; Eo, Eosinophil; Hb, Hemoglobin; Hct, Hematocrit; LDH, lactate dehydrogenase; Lymph, lymphocytes; Mono, Monocyte; PLT, Platelet; PT, Prothrombin Time; PT‐INR, Prothrombin Time‐International Normalized Ratio; RBC, Red blood cell; seg, Segment cell; T‐Bil, Total‐Bilirubin; TP, Total protein; WBC, White blood cell; Γ‐GTP, γ‐Glutamyl transpeptidase.

## 3 DISCUSSION

Here, we report a case of fulminant myocarditis that recovered with no neurological damage after a 38‐h period of asystole. Cardiac function in fulminant myocarditis is often reversible and improves after overcoming the acute phase, although patients who suffered cardiac arrest have a poor prognosis, even with ECMO or IABP.^9^, ^10^ There is also no established consensus on the reversibility of myocardial function, except in specific environments, such as cardiac surgery under cardiopulmonary bypass. A case of recovery after 20 h of asystole was reported previously; however, the present case suggests that patients with a longer duration of asystole could be saved if appropriate circulatory support is provided.

In fulminant myocarditis, temporary cardiopulmonary support is an important treatment approach as cardiac function can be restored following the acute phase.^2^, ^6^, ^11^, ^12^ An observational study assessing fulminant myocarditis found that ECMO is equivalent to ventricular assist devices (VAD) and easier to introduce.^9^, ^13^ Moreover, in fulminant myocarditis complicated by malignant ventricular arrhythmias, left VAD is unlikely to provide sufficient hemodynamic support when the right ventricle does not work effectively, whereas ECMO effectively bypasses biventricular failure. ECMO is the first treatment option for catastrophic myocarditis owing to low invasiveness, mobility for bedside implementation, and utility during cardiopulmonary resuscitation in cardiac arrests.^5^, ^14^

The concurrent use of devices such as VA‐ECMO with IABP or Impella, rather than VA‐ECMO alone, in patients with severe heart failure and cardiogenic shock may reduce the left ventricle load, minimize myocardial injury, and improve clinical outcomes.^15^ In severe cardiac dysfunction, the aortic valve does not open even when circulation is adequately secured by retrograde perfusion with VA‐ECMO, and left ventricular thrombosis is a concern.^16^ The combination of IABP and V‐A ECMO may reduce the risk of left ventricular thrombus by maintaining physiological antegrade blood flow.^9^ However, despite the theoretical advantage, the effectiveness of the combined use of IABP and V‐A ECMO is inconclusive.^17^ Several observational studies have shown conflicting results, and there are currently no randomized trials.^10^ In this case, the risk of ventricular thrombosis was significantly high when circulation was supported by V‐A ECMO only, as no antegrade blood flow was generated during asystole. Therefore, additional use of IABP might have been effective for preventing intraventricular thrombosis and reducing light ventricular load in the present case, which might result in the recovery of cardiac function. Regrettably, the current cause of fulminant myocarditis was not identified; however, this case illustrates that cardiac function might be restored even after prolonged asystole with adequate hemodynamic support.

## 4 CONCLUSION

We encountered a case of fulminant myocarditis that recovered with no neurological damage after a 38‐h period of asystole. Even if a patient with fulminant myocarditis develops asystole on ECG, there may still be a possibility of subsequent improvement.

## AUTHOR CONTRIBUTIONS

**Tomohiro Akutsu:** Writing – original draft. **Akira Endo:** Writing – review and editing. **Hiroyuki Sonobe:** Data curation; resources. **Keisuke Suzuki:** Data curation; resources. **Kiyoshi Murata:** Data curation; resources; supervision. **Yasuhiro Otomo:** Project administration.

## FUNDING INFORMATION

None.

## CONFLICT OF INTEREST STATEMENT

The authors have no conflict of interest to declare.

## ETHICS STATEMENT

The manuscript was approved by the Ethics Committee of Tsuchiura Kyodo General Hospital and Matsudo General Hospital.

## CONSENT STATEMENT

Written informed consent was obtained from the patient to publish this report.

## Data Availability

All data generated during this study can be accessed through direct communication with the corresponding author and through the agreement of all research team members.
